# A cold-atom Ramsey clock with a low volume physics package

**DOI:** 10.1038/s41598-024-51418-8

**Published:** 2024-01-09

**Authors:** A. Bregazzi, E. Batori, B. Lewis, C. Affolderbach, G. Mileti, E. Riis, P. F. Griffin

**Affiliations:** 1 SUPA and Department of Physics, University of Strathclyde, Glasgow, G4 0NG UK; 2 Institute of Physics, Laboratoire Temps-Fréquence, University of Neuchâtel, Avenue de Bellevaux 51, 2000 Neuchâtel, Switzerland

**Keywords:** Atomic and molecular physics, Applied physics, Quantum physics

## Abstract

We demonstrate a Ramsey-type microwave clock interrogating the 6.835 GHz ground-state transition in cold $^{87}$Rb atoms loaded from a grating magneto-optical trap (GMOT) enclosed in an additively manufactured loop-gap resonator microwave cavity. A short-term stability of $1.5 \times 10^{-11} \tau ^{-1/2}$ is demonstrated, in reasonable agreement with predictions from the signal-to-noise ratio of the measured Ramsey fringes. The cavity-grating package has a volume of $\approx$67 cm$^{3}$, ensuring an inherently compact system while the use of a GMOT drastically simplifies the optical requirements for laser cooled atoms. This work is another step towards the realisation of highly compact portable cold-atom frequency standards.

## Introduction

Compact frequency standards based on the interrogation of both ions and neutral atoms continue to receive much interest, with many different schemes now reported in the literature^1–10^. While compact clocks based on thermal atomic vapours and coherent population trapping (CPT) remain unrivalled in terms of size, weight and power (SWaP) they typically exhibit stabilities that are limited in the medium to long-term due to light-shift effects^11^ and the buffer gasses required to reduce atomic collisions^1^. In addition to this, CPT clocks struggle to reach atomic shot noise due to the limited signal-to-noise ratio (SNR), a consequence of the low detected signal photon count per atom. This limitation often requires complex interrogation schemes to optimise the clock stability^12–14^.

The development in the last decade of pulsed optically pumped (POP) clocks in thermal vapours has driven new research, with state-of-the-art stabilities^2,15,16^. However, these clocks still suffer from buffer gas shifts, providing the ultimate limitation to their long-term stabilities. Achievable Ramsey times within these systems are also limited to a few ms by spin relaxation of the thermal atoms^2,17^, or by the evolution time in an atomic beam^10^, restricting the short-term stability performance.

In an effort to combat these limitations several groups have now developed compact cold atom microwave clocks based on spherical optical-integrating sphere cavities^18,19^, cylindrical cavities^20,21^ and more recently loop-gap-resonator cavities^22^. Three examples of cold atom microwave clocks are now even commercially available^23–25^. Different laser cooling schemes with varying optical geometries have been used within these systems, with isotropic cooling of an optical molasses^18,19,21,23^, pyramid MOTs^26^ and larger traditional 6-beam MOTs^20,22^ all being utilised.

**Figure 1:**
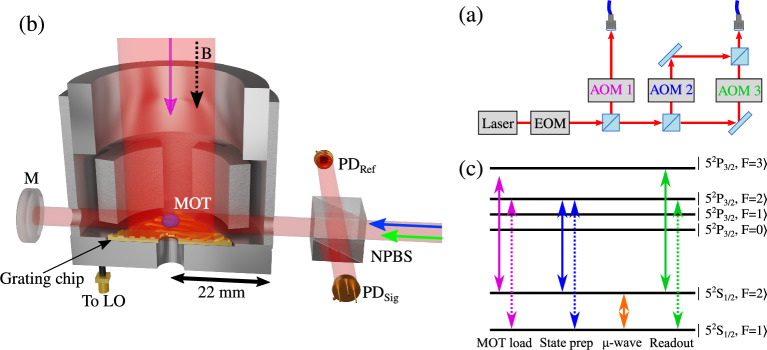
(**a**) Simplified optical set-up. AOM label text colours corresponds to the optical transitions shown in (**c**). (**b**) Schematic of the cavity physics package. Trap light (purple arrow) propagates parallel to the magnetic bias field (black dashed arrow), 90:10 non-polarising beam splitter (NPBS) splits the optical pumping (blue arrow) and readout (green arrow) light onto a reference photodiode ($\text {PD}_\text {Ref}$) and signal photodiode ($\text {PD}_\text {Sig}$) after being retro-reflected by a mirror (M). Local oscillator (LO) is connected via a SMA vacuum feedthrough. (**c**) Optical and microwave transitions used in the experiment. Solid arrows indicate light carrier frequency, dashed arrows show repump frequencies derived from the EOM.

In this letter we demonstrate operation and first stability measurement of a cold-atom atomic clock based on an additively manufactured loop-gap resonator (LGR) cavity with a volume of $\approx 67$ cm$^{3}$ incorporating an integrated GMOT^27^. As discussed by Affolderbach et al.^28^, the cavity geometry is the same whether it was machined or additively manufactured. However, additive manufacturing provides a more scalable and cost-effective means to reliably manufacture the relatively complex LGR structure in a highly repeatable manner, which would benefit future scalability of the device.

Although vapour-cell atomic clocks based on LGR structures with external diameters of order 1 cm have been demonstrated^17^, such small devices will have limited benefit when using cold atoms. Previous investigations with both GMOTs^29^ and 6-beam MOTs^30^ have shown that by constraining the trap beam diameter the maximum trapped atom number is limited, therefore constricting the potential stability of the final clock.

## Methods

A commercial laser system at 780 nm with an integrated electro-optic modulator (EOM) for the creation of optical sidebands on the carrier frequency is used throughout. The laser light is split into three distinct optical paths, each with a double passed acousto-optic modulator (AOM) for power and frequency control to enable trapping (AOM 1), state preparation (AOM 2) and state readout (AOM 3). A simplified schematic of the optical system is presented in Fig. 1a. The trapping light is coupled into an optical fibre to be passed to the physics package. The state preparation and state readout beams are coupled into a single additional fibre and likewise passed to the physics package.

The microwave cavity itself, described in detail in Ref^31^ consists of a loop-gap structure with a four electrode geometry and has an outer radius of 44 mm and a height of roughly 44 mm. The cavity operates in a $\text {TE}_{011}$-like mode, tuned to the ground-state-splitting of $^{87}$Rb with a quality factor of Q$\approx 360$ and is mounted within a stainless steel vacuum chamber, maintained at ultra-high-vacuum (UHV) by an ion pump. The relatively modest Q-factor of the cavity reduces cavity pulling effects, helping to improve the potential long-term stability of the device^31^. Optical access is enabled via viewports in the vacuum chamber along two orthogonal axes. The first axis is parallel to the cylindrical symmetry axis of the cavity (in the following this is referred to as the cavity axis) and allows the trap light to be directed onto the grating chip after expansion and collimation from the fibre. State preparation and state readout light, expanded from the fibre to a 1/e$^{2}$ diameter of 7 mm, is directed onto the atoms through two 4 mm holes drilled in the side of the cavity body. A retro-reflecting mirror for this light is placed outside the vacuum chamber to decrease the acceleration experienced by the atoms when interacting with the state preparation and probe beams and increase signal amplitudes. A simplified schematic of this is shown in Fig. 1b. No significant degradation of the cavity field is observed by the introduction of the holes in the cavity body^31^.

A pair of anti-Helmholtz coils are mounted within the vacuum chamber, along the cavity axis in order to create the quadrupole magnetic field required for the trapping process. Three orthogonal pairs of Helmholtz shim coils, mounted externally to the vacuum chamber are used for the cancellation of external stray DC magnetic fields and to apply a magnetic bias along the cavity axis of $\approx 100$ mG in order to lift the atomic degeneracy during optical molasses and clock interrogation. We note that the current demonstration is a proof of concept with no magnetic shielding of the experiment present, limiting the potential stability of the system.

The experimental cycle is initiated by turning the trapping coils on with the trap light tuned to be approximately $\Delta$=-2$\Gamma$ red detuned ($\Gamma /2\pi$=6.07 MHz) from the $^{87}$Rb $\text {D}_{2}$ *F*=2$\rightarrow$*F’*=3 cycling transition, with re-pump light generated by the EOM operating at 6.57 GHz to produce 5% optical sidebands. A Rb vapour, maintained at the $1\times 10^{-9}$ Torr level, is produced by resistively heating a pair of alkali metal dispensers. We then perform a 6 ms optical molasses by turning the trap coils off and linearly ramping the light detuning to $\Delta$=-5$\Gamma$ while simultaneously decreasing the trap light intensity. After molasses we measure atomic temperatures of $\approx 10~\mu K$. In order to decrease the clock cycle time and mitigate experimental dead time, we employ atom recapture between experimental cycles^3,32^. In steady state this allows the trapping of $>3\times 10^6$ atoms with a load time of 100 ms for a clock cycle operating at $\approx$7 Hz. Once the atoms have been trapped and cooled, the trap light is extinguished by AOM 1 and blocked by a mechanical shutter^33^ to ensure no light leakage during microwave interrogation. After molasses, the atoms are assumed to be roughly evenly distributed between the five $m_F$ levels of the $F=2$ hyperfine ground-state manifold. We therefore employ a 1 ms optical pumping stage with 10 $\mu$W total power of linearly polarised light, polarisation axis parallel to the quantization axis, tuned to the *F*=2$\rightarrow$*F’*=2. The repump frequency is also simultaneously changed to 6.83 GHz to resonantly drive the *F*=1$\rightarrow$*F’*=2 transition. Due to selection rules this pumps $>95\%$ of the atoms into the 5$^{2}\text {S}_{1/2}$, $\mathinner {|{{F=2,m_F=0}}\rangle }$ clock state^34^ as seen by the almost complete elimination of the $m_F=1\rightarrow m_F'=1$ transitions, when scanning the microwave detuning, increasing the contrast of the resulting clock signal. A diagram of all atomic transitions utilised in the experiment is shown in Fig. 1c

**Figure 2:**
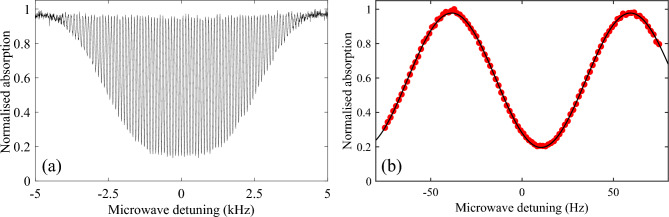
(**a**) Typical Ramsey fringe taken with a 10 ms free evolution time. (**b**) Central Ramsey fringe obtained at 10 ms free evolution time. Red points indicate measured normalised probe absorption while the black line shows a sinusoidal fit to the data. The fringe offset from 0 Hz detuning corresponds to the frequency shift expected from the second-order Zeeman shift at a 100 mG bias field.

A Keysight E8257D microwave synthesizer is used as a local oscillator and microwave source throughout. Square microwave $\pi /2$ pulses with a duration of 200 $\mu$s and typical power levels of 0.04 mW are applied to the atoms. The specified phase noise performance of the local oscillator allows the Dick effect^35^ to be reduced to the $6.12\times 10^{-13}$ level at 1 s for the experimental cycle time. After the desired microwave pulses have been applied, the atomic states are read out by an absorption method using 30 $\mu$W of optical power, measured by recording the transmission on a photodiode of two subsequent probe pulses. First a short pulse of readout light tuned to the *F*=2$\rightarrow$*F’*=3 transition is applied, giving an measure of the number of atoms in the $F=2$ ground-state. We then apply a second readout pulse with re-pump light present. This measures the total number of atoms, providing a normalisation of the signal and reduced sensitivity to atom number fluctuations. Intensity noise in the readout pulses is reduced by the use of a reference photodiode.

## Results

**Figure 3:**
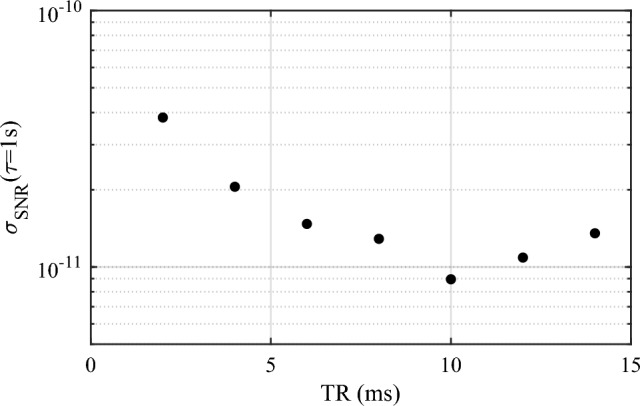
Predicted short-term stability as a function of Ramsey time.

An example Ramsey fringe taken with a Ramsey time, $T_R$ = 10 ms, corresponding to a fringe linewidth of 50 Hz is shown in Fig. 2. This fringe exhibits an SNR of around 110, measured at the half-width points of the central fringe, where SNR is defined as the ratio of the peak amplitude divided by the amplitude noise. The predicted SNR limited short-term relative frequency instability of a local oscillator stabilised to an atomic transition in terms of Allan deviation is given by^36^:

1 $$\begin{aligned} \sigma _{SNR}(\tau )=\frac{1}{\pi C}\frac{\Delta f}{f_0}\frac{1}{SNR}\sqrt{\frac{T_C}{\tau }} \end{aligned}$$

where *C* is the fringe contrast, $\Delta f$ is the signal linewidth ($\approx 1/2T_R$), $f_0$ is the central frequency (6.8346... GHz), $T_C$ is the full experimental cycle time ($T_C=140$ms) and $\tau$ is the averaging time. We use this relationship as a basis to optimise our experimental cycle to maximise the potential stability of our clock. A plot of the predicted stability, using the measured SNR, as a function of the Ramsey time is shown in Fig. 3. From this we find that as the Ramsey time is increased the predicted stability also improves up to the level of $8.95\times 10^{-12}$ for a 10 ms Ramsey time. After this time however the stability begins to decrease as the SNR is degraded. This degradation in SNR is attributed to atomic losses due to both the thermal expansion of the cloud and the atoms falling out of the probe region under gravity. The extension of this Ramsey time should be possible by moving the holes in the cavity body lower down, introducing elliptical holes to maintain good probe-atom overlap along the path of gravity or by implementing a grating-chip atomic fountain^37^. This last option is particularly attractive because as with traditional atomic fountains it would be possible to apply both $\pi /2$ pulses when the atoms are at the same vertical point of the cavity. This will allow the phase difference observed by the atoms between the two microwave pulses to be minimised, essential for high contrast Ramsey fringes in a relatively low-Q cavity such as used here. A grating-chip atomic fountain (using CPT interrogation) such as this has already demonstrated Ramsey times out to 100 ms, with a corresponding fringe linewidth of 5 Hz^37^. The centre-of-mass of the atomic cloud in this case would be the same for both microwave pulses. However, longer Ramsey times will result in a larger atomic cloud due to its ballistic expansion during the time of flight. For a temperature of 4 $\mu$K, the cloud size would increase from a 2$\sigma$ diameter of 1 mm to 4 mm over the projected 100 ms Ramsey time. As a consequence, the microwave field will be sampled over a larger volume for the second pulse, increasing the field inhomogeneity experienced by the atoms^31^. This will not pose too great an issue; even a 30% field inhomogeneity, a reasonable worst-case assumption based on similar previous LGR cavities^4^, only results in a reduced maximum contrast at the 80% level^31^. Substituting this new contrast into Eq. (1) along with the reduced atomic linewidth still results in an eight-fold improvement to the short-term stability.

**Figure 4:**
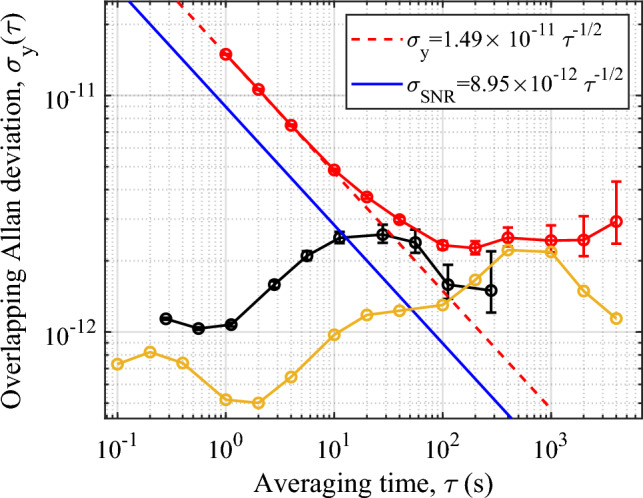
Red points: Overlapping Allan deviation of local oscillator’s stability when locked to atomic signal. Red dashed line: $\tau ^{-1/2}$ dependence of the measured 1 s stability. Blue solid line: stability predicted by (1) and measured fringe SNR. Black points: stability limit due to the second-order Zeeman shift. Yellow points: measured GPSDO reference oscillator stability. Error bars represent 1$\sigma$ confidence bound.

To assess the stability of our system we stabilise the local oscillator to the atomic signal. The signal is modulated and demodulated at the fringe’s half maximum to construct the error signal which is used to feedback onto the local oscillator by voltage tuning its 10 MHz internal reference. Figure 4 shows an overlapping Allan deviation of the resulting frequency stability when compared to an oven controlled quartz crystal oscillator (Wenzel Associates 501-29647) disciplined to GPS (GPSDO). From Fig. 4 we see that the clock stability averages down with a $1.5\times 10^{-11}~\tau ^{-1/2}$ dependence out to 10 s, at which point it deviates slightly from the ideal $\tau ^{-1/2}$ dependence. This is in reasonable agreement with the theoretical stability obtained from Fig. 3 at a 10 ms Ramsey time and is well above the ultimate stability limit set by the quantum projection noise (QPN) of $4.9\times 10^{-13}~\tau ^{-1/2}$, calculated by replacing the SNR term in (1) by $\sqrt{N}$, where *N* is the atom number.

As the experiment is currently operated in a magnetically unshielded environment the instability contribution due to the second-order Zeeman shift was expected to limit the clock stability in the medium-term. This was confirmed by measuring the magnetic field stability via the $\mathinner {|{F=1, m_F=1}\rangle } \rightarrow \mathinner {|{F'=2, m_F'=1}\rangle }$ microwave transition, exhibiting a magnetic field sensitivity of $\beta _1$=1.4 MHz/G^38^. The expected stability of the $m_F=0$ clock transition ($\beta _0$=575 Hz/G$^{2}$^38^) could then be calculated, shown as black points in Fig. 4. This plot shows that the second-order Zeeman shift is indeed an important limitation to the clock stability at $\tau>$10 s, due to a pronounced hump in the magnetic field stability around this time, after which the stability averages down slightly to the level of $<2\times 10^{-12}$. For future iterations of this set-up it will be imperative to introduce magnetic shielding for long-term stability performance.

Another limiting factor to the clock stability in the medium term is the GPSDO reference. Three-corner-hat stability measurements of this reference against two commercial Cs beam clocks (OSA 3235B and Microchip 5071A) indicates excellent short-term performance of the GPSDO $<1\times 10^{-12}$. At 700 s however, a peak in the instability is observed at $\approx 2\times 10^{-12}$. This peak is associated with the time constant of the tuning loop used to reference the oscillator to the GPS signal. When taken in conjunction with the stability limit due to the Zeeman effect, these two effects help explain the flattening of the clock stability for averaging times $>100$ s.

To more fully characterise the short-term stability ($\tau$=1 s) of the system a stability budget of the main instability contributions to the clock was constructed, shown in Table 1. The short-term clock performance is found to be primarily limited by the Ramsey fringe SNR, discussed above. In future we expect to be able to improve the SNR contribution to short-term stability towards the projection noise limit. This will be achieved by increasing the Ramsey time, optimisation of the optical detection process and suppression of intensity and frequency fluctuations in the probe beam.

**Table 1:** Table of noise sources and their contributions to the 1 s Allan deviations stability.

Noise source	$\sigma$ contribution $(\tau =1s)$
SNR	$8.95 \times 10^{-12}$
Electronic noise	$1.14 \times 10^{-12}$
Dick	$6.12 \times 10^{-13}$
Zeeman	$1.07 \times 10^{-12}$
QPN	$4.90 \times 10^{-13}$
Total $\bigg (\sqrt{\sigma _{SNR}^2+\sigma _{Electronic}^2+\sigma _{Dick}^2}\bigg )$	$9.04\times 10^{-12}$
Measured	$1.49\times 10^{-11}$

The next largest contributor to the 1 s clock stability after the fringe SNR is electronic noise on the voltage line used to tune the 10 MHz local oscillator. While this is not a limiting factor to the clock stability at present, careful minimisation of this noise source will be required when moving towards a magnetically shielded experiment where the expected clock stability should improve below the $1\times 10^{-12}$ level. The total predicted short-term stability from Table 1 is found to be  1.6 times better than the measured stability. Estimates for systematic errors arising from the microwave cavity, such as cavity pulling and phase inhomogeneities, in addition to those from Stark shifts, have contributions are of order $10^{-14}$ or lower. Efforts are ongoing to identify the remaining instability contributions in order to minimise them in future iterations of the set-up.

## Conclusion

In conclusion, we have demonstrated a compact integrated atomic package, which incorporates a grating-MOT and an additively manufactured loop-gap-resonator microwave cavity, for a cold-atom microwave clock showing high-contrast Ramsey fringes for $T_R>$10 ms. In the present system the experimentally optimised short-term clock stability is measured as $\sigma _y(\tau )=1.5\times 10^{-11}~\tau ^{-1/2}$, primarily limited by the Ramsey fringe SNR. In the medium-term the clock stability is primarily limited by the second-order Zeeman shift due to operating in an unshielded environment. A secondary limit is placed on the medium-term stability performance by the reference oscillator itself exhibiting a stability bump at around 700 s. By addressing these issues, we expect to improve the short-term stability to be more in-line with other cold-atom cavity clock demonstrations^18,22–24^.

Thermal-vapour clocks are subject to collision shifts. While there has been a heroic work on minimising this effect through the use of buffer gas mixtures with opposite shifts and careful control of thermal effects, the clock system is still sensitive to low-frequency drifts from environmental effects. The time scale over which these effects become relevant will depend on the specific implementation but are typically on the scale on hours to days. The use of laser-cooled atoms in high vacuum removes these effects. However, careful design is still required to remove other environmental effects, such as on laser intensity and frequency, and magnetic field noise. A next generation of the system will include these considerations to demonstrate the expected long-term performance.

The current physics package is inherently compact and remains highly amenable to further miniaturisation. The use of additive manufacturing also maintains a highly scalable manufacturing process. Additively manufactured vacuum chambers have also recently demonstrated compatibility with UHV^39^ pressure levels. This raises the enticing possibility of the cavity body itself simultaneously acting as a UHV chamber, allowing the entire physics package to be manufactured as a single bulk component, drastically reducing the size of the system. Additive manufacturing in this instance should offer a reproducible and cost-effective means of engineering the physics package in a highly scalable fashion. Initially we envisage an actively pumped system with a physics package volume (not including magnetic shielding) of $\approx$100 cm$^{3}$ being an achievable target. Passively pumped vacuum chambers have now been shown to maintain the UHV levels required for atom trapping for extended periods^40,41^, allowing for a reduction in power consumption by negating the continuous use of an ion pump. Whilst the stability performance of our clock is currently 10-50 times below the state-of-the-art commercial offerings^23–25^, similar performances should be possible with the discussed improvements to the system. With more focus on system integration in a second generation of the clock, the experiment size should also be much improved, in line or even smaller than current offerings due to the highly compact physics package. We therefore believe this work represents a step towards highly compact and portable cold-atom frequency standards.

## Data Availability

The data that support the findings of this study are available from 10.15129/bb0d3614-6394-4d0e-8649-b1bf66c71722.
